# Shot Noise analysis of subthreshold membrane potential activity in neurons

**DOI:** 10.1186/1471-2202-14-S1-P108

**Published:** 2013-07-08

**Authors:** Marco Brigham, Alain Destexhe

**Affiliations:** 1 UNIC, CNRS, Gif-sur-Yvette, 91198, France

## 

Shot noise is one of the most extensively harnessed phenomena used to model different aspects of neural activity in neuroscience. Shot noise processes [1] display very characteristic fluctuations that correspond to the sum of causal system responses to unitary event arrivals that follow a Poisson distribution.

In general, applications of shot noise [2-4] have been confined to the so-called *Campbell Limit *corresponding to steady-state regime, or to specific post-synaptic responses that make the process Markovian or with the assumption of homogeneous Poisson event arrivals. In this work we go beyond this context to show that the shot-noise formalism can be used to analyze the synaptic activity of neurons beyond the Campbell limit, for any type of bounded post-synaptic response function and non-homogeneous Poisson event arrivals.

This enhancement of technique allows for two main applications.

1) Computation of the full density for current synapses and statistical moments for conductance synapses. This yields a statistical description of the subthreshold membrane potential. This includes considering distributions on the afferent populations over biological parameters such as peak intensity, number of independent channels, or dynamical parameters such as distribution of incoming firing rate or variable incoming firing rate. The picture obtained is of particular interest for various applications such as calculating the transfer function.

2) Under stationarity assumptions, this technique offers the potential to reverse the moments of membrane potential integration. This offers a candidate method to infer characteristics of the synaptic activity (and therefore of the network activity) from the analysis of the subthreshold variations of the membrane potential. This application is of particular interest for the analysis of intracellular recordings *in vivo*.

We illustrate these techniques by comparing analytic expressions with numerical estimates, as well as to neurons subject to a large range of synaptic activity (including low release rate far from the diffusion limit).

In conclusion, due to their flexibility as a modeling tool, shot noise analysis appears as a very useful tool in computational neuroscience to model post-synaptic and sub-threshold membrane potential responses to incoming network activity, and also as a tool for electrophysiological analysis of network activity from intracellular recordings.
